# ﻿ *Chicosa* gen. nov. (Araneae, Lycosidae), a new genus of wolf spiders from East Asia

**DOI:** 10.3897/zookeys.1260.161209

**Published:** 2025-11-13

**Authors:** Ze-Hong Tao, Dan Fu, Chang-Jun Wu, Yang Wang, Li-Juan Liu, Yu-Fa Luo

**Affiliations:** 1 Key Laboratory of Wetland Biodiversity of the Jianhu Basin of Shaoxing, School of Life and Environmental Sciences, Shaoxing University, Shaoxing 312000, China Shaoxing University Shaoxing China

**Keywords:** Araneae, Lycosidae, mitochondrial genes, morphology, new combination, new genus, phylogenetics

## Abstract

The new genus *Chicosa***gen. nov.** (type species: *Alopecosa cinnameopilosa* (Schenkel, 1963) is described and the new combination *Chicosa cinnameopilosa* (Schenkel, 1963), **comb. nov.** is made. Phylogenetic analyses of mitochondrial loci do not place *Chicosa cinnameopilosa* into *Alopecosa* or the subfamily Lycosinae but rather suggest it is sister to *Wadicosa* Zyuzin, 1985 + *Pardosa* C. L. Koch, 1847. Descriptions of the new genus and known species, supplemented by photographs, a distribution map, and phylogenetic evidence, are provided.

## ﻿Introduction

The family Lycosidae (wolf spiders) represents one of the most species-rich groups within the order Araneae, and its taxonomic classification has long been a focal yet challenging subject in arachnological research. The genus-level classification of Lycosidae has long relied on integrated morphological characters, including the male palpal organ, body striation patterns, and leg spine arrangements. However, certain species in East Asia may exhibit generic misplacements due to historically broad classification criteria or incomplete information or a lack of redescriptions of type specimens (Xu 2010; Liu et al. 2025).

*Alopecosa* Simon, 1885, a species-rich lineage within Lycosidae, currently comprises 172 recognized species, ranking as the third largest genus in the family (World Spider Catalog 2025). Nevertheless, the genus has long been taxonomically contentious, exhibiting significant regional discrepancies in species composition, and is widely regarded as a wastebasket taxon (Wang et al. 2025). Marusik and Kovblyuk (2011) noted that the morphological disparities between some *Alopecosa* species and the type species exceeded generic-level variation, suggesting that the genus is likely polyphyletic and requires revision and division into multiple independent genera. Despite over a century of research, regional limitations in early studies and a lack of international collaboration have perpetuated numerous misidentifications and synonymies. More critically, the current classification system often accommodates species with morphological traits that deviate from established generic diagnoses, a practice that has compromised the accuracy of phylogenetic reconstructions (Framenau 2015; Esyunin and Ponomarev 2018; Naumova and Deltshev 2023).

Examination of specimens from several provinces of China (Hebei, Inner Mongolia, Liaoning, and Shandong) revealed significant taxonomic discrepancies between both sexes of *Alopecosa cinnameopilosa* (Schenkel, 1963) and the type species *Alopecosa fabrilis* (Clerck, 1757). Molecular phylogeny also suggested the significant divergence of this species from *Alopecosa*. To resolve these inconsistencies, we establish a new genus, thereby refining the classification of Lycosidae. Comprehensive morphological descriptions and illustrations are provided for the new taxon.

## ﻿Material and methods

### ﻿Morphological treatment

All specimens were preserved in 95% ethanol. The specimens were examined, expounded, photographed, and measured using a Phenix stereomicroscope. Palps and epigynes were examined and described after dissection using a Sony digital Camera and Wemacro software. Epigynes were cleared by immersing them in pancreatin (Álvarez-Padilla and Hormiga 2007). Images were focus-stacked using the software Helicon Focus ver. 3.10. Scanning electron microscope (SEM) microphotographs were obtained with a Zeiss SIGMA300 SEM (preparation procedures as in Xiao and Li 2012). Eye sizes were defined as the maximum dorsal diameter. Leg measurements are given as: total length (femur, patella and tibia, metatarsus, tarsus). All measurements are reported in millimeters (mm).

The following abbreviations are used in the text and figures: ALE-anterior lateral eye; AME-anterior median eye; PLE-posterior lateral eye; PME-posterior median eye.

Specimens examined here are deposited in the Key Laboratory of Wetland Biodiversity of the Jianhu Basin of Shaoxing, School of Life and Environmental Sciences, Shaoxing University, Shaoxing, China.

### ﻿DNA extraction, amplification and sequencing

Genomic DNA was extracted from two legs of the paratype (LCG_11) with the TIANamp Genomic DNA Kit (Tiangen, Beijing, China). Partial sequences of the mitochondrial *COI*, 12S rRNA, and 16S rRNA genes were amplified using the primer pairs: LCO1490/HCO2198 for *COI* (Folmer et al. 1994); 12Sai/12Sbi for 12S rRNA (Kocher et al. 1989); and 16Sar/16SB2 for 16S rRNA (Tan et al. 1999). PCR reactions (25 μl volume) contained: 12.5 μl MyFi Mix (Bioline, USA), 1 μl each primer (10 μM), 2 μl DNA template, and 8.5 μl ddH_2_O. For *COI* amplification, the PCR protocol was as follows: initial denaturation at 94 °C for 5 min; 40 cycles of 94 °C denaturation (30 s), 45 °C annealing (90 s), 72 °C extension (1 min); and final extension at 72 °C for 5 min. For 12S rRNA amplification, the PCR protocol was as follows: an initial denaturation at 94 °C for 1 min; 35 cycles of 94 °C denaturation (25 s), 49 °C annealing (30 s), 72 °C extension (20 s); and a final extension at 72 °C for 6 min. For 16S rRNA amplification, the PCR protocol was as follows: initial denaturation at 94 °C for 1 min; 35 cycles of 94 °C denaturation (30 s), 49 °C annealing (45 s), 72 °C extension (1 min); and a final extension at 72 °C for 5 min. All PCR products were purified and directly sequenced via primer walking using BigDye technology on an ABI 3730 automated sequencer (Applied Biosystems, Foster City, CA, USA) at Tsingke Biotechnology Co., Ltd., Hangzhou, China. New sequences were submitted to GenBank (Suppl. material 1).

### ﻿Phylogenetic reconstruction

Each gene was aligned using MAFFT ver. 7.0, applying the G‐INS‐i algorithm for highly conserved sequences (*COI*) and Q‐INS‐i for sequences with more variable regions (12S rRNA and 16S rRNA). Ambiguously aligned sites/regions were eliminated using Gblocks (Castresana 2000). We first used the concatenated *COI*, 12S rRNA and 16S rRNA dataset (Suppl. material 2) of 329 wolf spider species, including the sequences of 328 species downloaded from GenBank and BOLDSYSTEM (Suppl. material 1), to construct the maximum likelihood (ML) tree in W-IQ-TREE (Trifinopoulos et al. 2016). The FreeRate heterogeneity and Bayesian Information Criterion identified the best partition-specific substitution model for each gene partition (GTR+F+R6 for *COI*; GTR+F+I+G4 for 12S rRNA; TIM2+F+I+G4 for 16S rRNA). Perturbation strength (p) and number of iterations since the last optimal tree discovery (c) were specified as 0.5 and 1000, respectively. Branch support was assessed via ultrafast bootstrap (UFBoot; Minh et al. 2013) and SH-aLRT (Guindon et al. 2010) with 1000 maximum replicates and a minimum correlation coefficient threshold of 0.99. The outgroups include *Pisaura ancora* Paik, 1969, *P. lama* Bösenberg & Strand, 1906 and *P. mirabilis* Clerck, 1757. Second, we reconstructed the ML tree of 85 species of Lycosinae + Pardosinae using concatenated *COI*, 12S rRNA and 16S rRNA sequences (Suppl. material 3) with the outgroups *Hippasa pantherine* Pocock, 1899, *H. deserticola* Simon, 1889 and *H. madraspatana* Gravely, 1924. This analysis was also performed in W-IQ-TREE with the optimal substitution models (GTR+F+I+G4 for *COI*; TIM2+F+I+G4 for 12S rRNA; and TIM2+F+R3 for 16S rRNA) using the same sets as above.

## ﻿Taxonomy

### ﻿Family Lycosidae Sundevall, 1833

Common name 狼蛛科

#### ﻿ Chicosa gen. nov.

Taxon classificationAnimaliaAraneaeLycosidae

9E712250-A451-50FB-B15B-3F9C2B1ED9F8

https://zoobank.org/8E68A01E-D4F4-41D1-9588-2F206D3E2562

##### Type species.

*Alopecosa cinnameopilosa* Schenkel, 1963.

##### Etymology.

The generic name *Chicosa* is a combination of the Mandarin Pinyin chi (from 螭, chī, a hornless dragon in Chinese mythology) and the common lycosid suffix -cosa (from Greek kósos, meaning ‘creature’). The gender is feminine.

##### Diagnosis.

**Figure 1.**
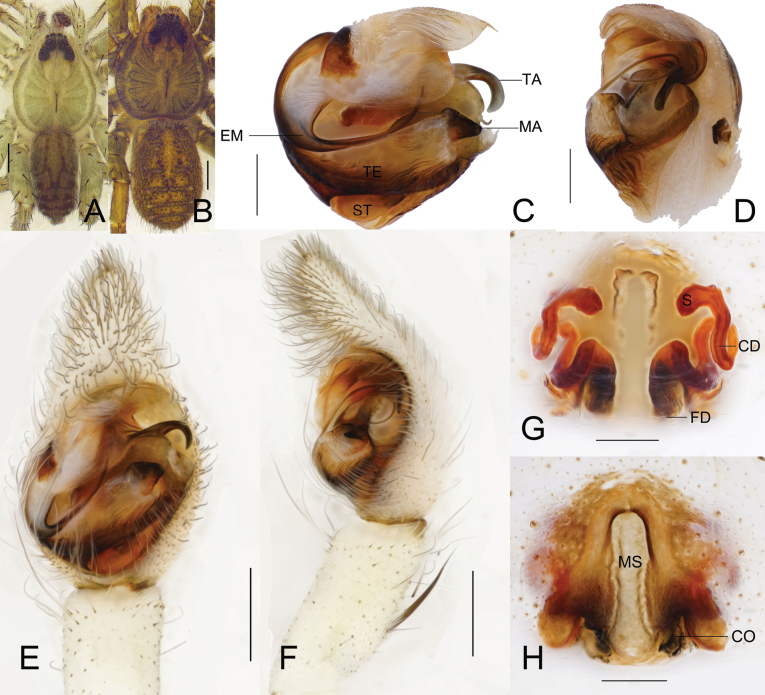
*Chicosa cinnameopilosa* (Schenkel, 1963), comb. nov. A. Male habitus, dorsal view; B. Female habitus, dorsal view; C. Left male pedipalp bulb, ventral view; D. Same, retrolateral view; E. Left male palp, ventral view; F. Same, retrolateral view; G. Epigyne, dorsal view; H. Same, ventral view. Scale bars: 1 mm (A, B); 0.4 mm (E, F); 0.2 mm (C, D, G, H). Abbreviations: CD = copulatory duct; CO = copulatory opening; EM = embolus; FD = fertilization duct; MA = median apophysis; MS = median septum; S = spermatheca; ST = subtegulum; TA = terminal apophysis; and TE = tegulum.

**Figure 2.**
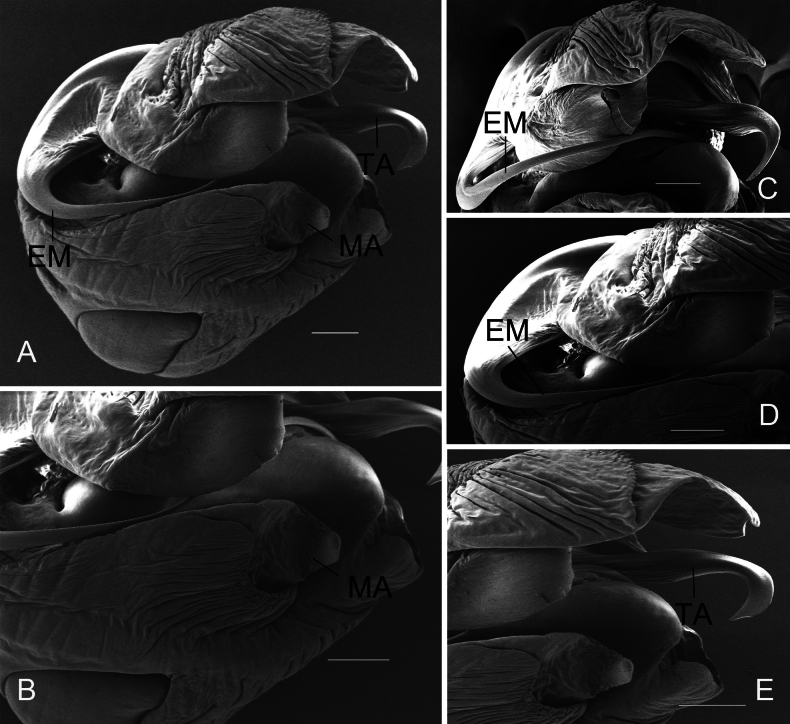
*Chicosa cinnameopilosa* (Schenkel, 1963), comb. nov. A. Left male pedipalp bulb, ventral view; B. Median apophysis, ventral view; C. Embolus, retrolateral view; D. Embolus, ventral view; E. Terminal apophysis and median apophysis, ventral view. Scale bars: 0.1 mm (A−E). Abbreviations: EM = embolus; MA = median apophysis; TA = terminal apophysis.

**Figure 3.**
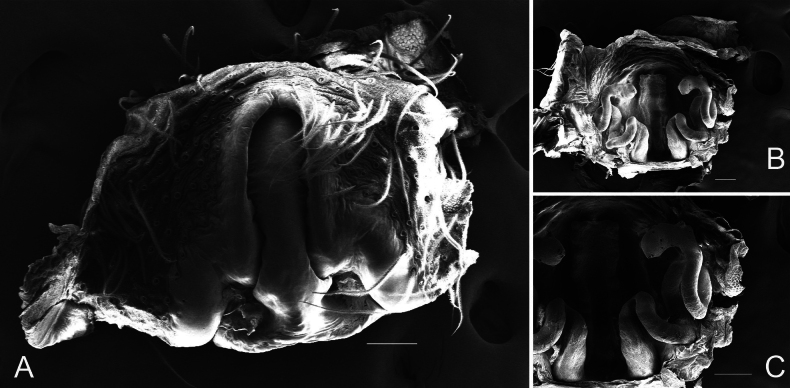
*Chicosa cinnameopilosa* (Schenkel, 1963), comb. nov. A. Epigyne, ventral view; B. Epigyne, dorsal view; C. Copulatory duct, dorsal view. Scale bars: 0.1 mm (A–C).

*Chicosa* gen. nov. is distinguished by the following combination of characters. Terminal apophysis (TA) well-developed, hook-shaped (Figs 1A, E, 2A, E); embolus (EM) originates low, extends transversely, then twists retrolaterally before curving to anterior part of bulb (Figs 1C, D, E, 2A, B, D); median apophysis (MA) undivided, cymbiform (Figs 1C, E, F, 2A, B, E). Median septum (MS) inverted T-shaped (Figs 1H, 3A); copulatory ducts (CD) extremely long, spirally coiled (Figs 1G, 3B, C). Differs from *Alopecosa* in: MA cymbiform and entire (vs. typically bipartite), TA hook-shaped (vs. often dentiform), EM basally originating (vs. non-basal), and CD extremely long and spiral (vs. shorter and simpler). Differs from *Pardosa* C. L. Koch, 1847 in: MA cymbiform and entire (vs. usually bipartite), TA hook-shaped (vs. dentiform), and EM basally originating (vs. non-basal). Although sharing two retromarginal cheliceral teeth with *Pardosops* Roewer, 1955, *Chicosa* exhibits a more complex palpal organ: MA cymbiform (vs. short and robust), TA elongate and hook-shaped (vs. two spinose processes). Differs from *Acroniops* Simon, 1898 in possessing four eyes (vs. two) in the anterior row. Distinguished from *Chorilycosa* Roewer, 1960 by the straight (vs. recurved) anterior eye row and hook-shaped TA (vs. two distinct spines). Differs from *Leimonia* C. L. Koch, 1847 in the straight anterior eye row with subequal width to second row (vs. strongly recurved and wider). Differs from *Draposa* Kronestedt, 2010 by the undivided, cymbiform MA with a single acute apex bent ventrolaterally (vs. MA transversely extended, with broad base bearing processes and narrow distal part bearing a small subapical projection), and the complete inverted T-shaped MS (vs. epigynal atrium only partially divided by a tongue-like septum). Differs from *Wadicosa* Zyuzin, 1985 in: retromargin with two teeth (vs. three), subtegulum small and positioned directly below tegulum (vs. large and anteroventrally shifted), TA without spiral torsion (vs. strongly twisted), and EM originating low and extending transversely (vs. originating antero-apically); female with distinct inverted T-shaped MS (vs. absent).

##### Phylogeny.

**Figure 4.**
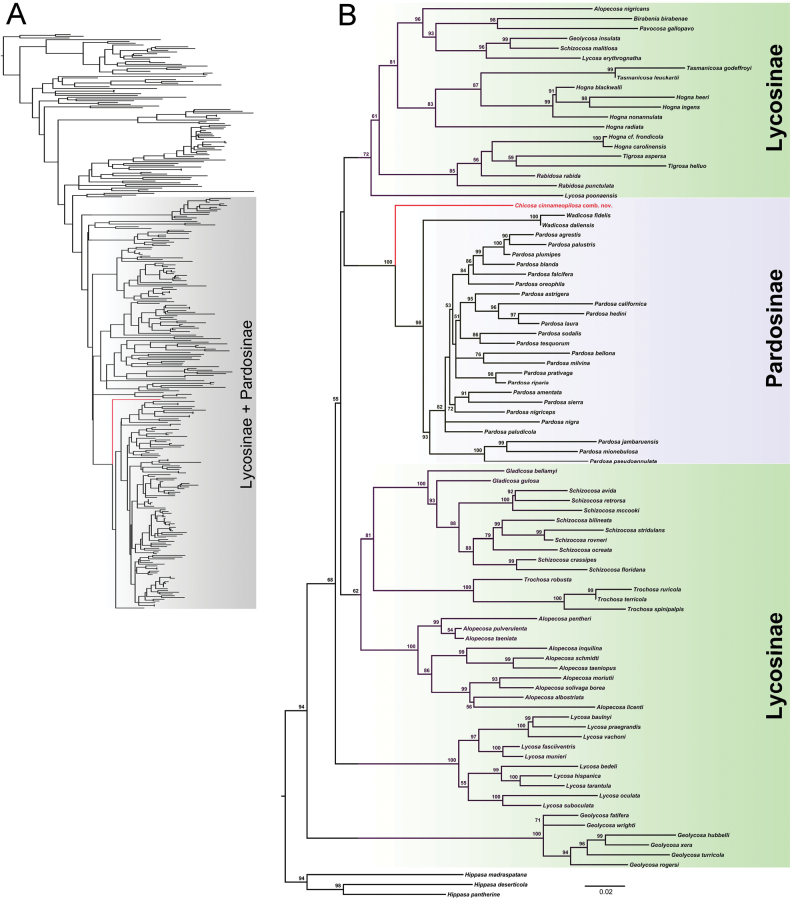
Phylogenetic trees reconstructed with the ML method. A. ML tree constructed using the concatenated *COI*, 12S rRNA and 16S rRNA dataset of 329 species of Lycosidae; B. ML tree reconstructed using the concatenated *COI*, 12S rRNA and 16S rRNA sequences of 85 species of Lycosinae and Pardosinae. The numbers at the nodes represent bootstrap support values from the ML analyses. *Chicosa cinnameopilosa* comb. nov. is shown in red.

*Chicosa cinnameopilosa* comb. nov. clusters together with the species of Pardosinae, and it is sister to *Wadicosa* + *Pardosa* (Fig. 4).

##### Description.

See species description.

##### Composition.

Only the type species.

##### Distribution.

**Figure 5.**
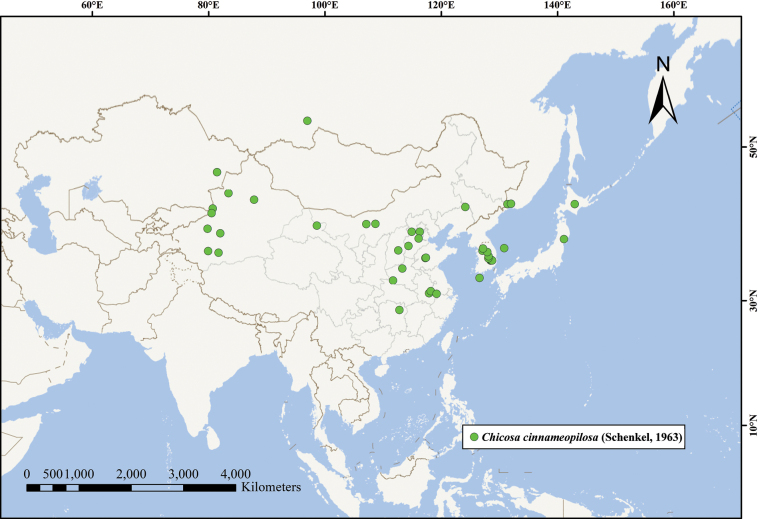
Geographic distribution of *Chicosa cinnameopilosa* (Schenkel, 1963).

**China** (Anhui, Beijing, Hebei, Henan, Hunan, Inner Mongolia, Jilin, Shanxi, Shandong, Xinjiang and Zhejiang provinces), Japan, Kazakhstan, North Korea and Russia (Fig. 5).

#### ﻿ Chicosa cinnameopilosa

Taxon classificationAnimaliaAraneaeLycosidae

(Schenkel, 1963) comb. nov.

90338645-3497-56DD-87F5-157A8596D6CE

Figs 1, 2, 3

Tarentula cinnameopilosa Schenkel, 1963: 333, fig. 192 (♀); Hu 1984: 246, fig. 258. 1–2 (♀♂).
Pardosa lusisi : Šternbergs 1981: 60, fig. 1 (♀♂).
Alopecosa cinnameopilosa : Li and Chen 1982: 66, figs 1–3 (♀♂); Song 1982: 76, figs 3–4 (♂); Zhu and Shi 1985: 129, fig. 113a–c (♀); Song 1986: 78, figs 19–22 (♀); Song 1987: 215, fig. 174 (♀♂); Tanaka 1987: 17, figs 1–4 (♀♂); Zhang 1987: 144, fig. 119.1–3 (♀♂); Paik 1988: 92, figs 25–34 (♀♂); Hu and Wu 1989: 186, fig. 155.1–5 (♀♂); Chen and Zhang 1991: 216, fig. 221.1–4 (♀♂); Tanaka 1992: 326, figs 13–16 (♀♂); Yin et al. 1997: 60, fig. 25a–f (♀♂); Song et al. 1999: 316, figs 186K, 187B (♀♂); Marusik et al. 2000: 77; Song et al. 2001: 231, fig. 141A–D (♀♂); Namkung 2002: 313, fig. 20.7a–b (♀♂); Namkung 2003: 315, fig. 20.7a–b (♀♂); Tanaka 2009: 238, figs 93–94 (♀♂); Zhu and Zhang 2011: 253, fig. 182A–D (♀♂); Yin et al. 2012: 791, fig. 395a–f (♀♂); Zhang et al. 2022: 123, fig. 87A–H (♀♂).

##### Material examined.

**China, Shandong**: • 2♂ 3♀, Taian City, Taishan District, Xujiazhuang village, 36°10'20"N, 117°15'17"E, elev. 135.4 m, 3 August 2023, L.J. Liu and Y. Cheng leg. • 1♂ 2♀, Taian City, Daiyue District, Yunbeishan village, 36°12'32"N, 117°20'42"E, elev. 141.5 m, 4 August 2023, L.J. Liu and Y. Cheng leg. **Liaoning**: • 22♀, Tieling City, Changtu County, Hongshan village, Hongshan Reservoir, 42°52'55"N, 124°07'24"E, elev. 150.7 m, 1 August 2023, L.J. Liu and Y. Cheng leg. **Hebei**: • 1♂ 1♀, Shijiazhuang City, Yuanshi County, Panlong Lake, 37°47'47"N, 114°21'44"E, elev. 112.8 m, 16 July 2023, L.J. Liu and Y. Cheng leg. **Inner Mongolia**: • 6♀, Bayannur City, Urad Front Banner, Shunda Petroleum Gas Station, 40°43'32"N, 108°41'4"E, elev. 1054.2 m, 13 August 2024, Z.H. Tao and H.F. Shi leg. • 2♀, Bayannur City, Hanggin Rear Banner Railway Station, 40°41'46"N, 107°6'53"E, elev. 1035.5 m, 20 August 2024, Z.H. Tao and H.F. Shi leg.

##### Diagnosis.

See generic diagnosis.

##### Description.

**Male** (Fig. 1A): Total length 7.15. Carapace 3.80 long, 3.15 wide; opisthosoma 3.28 long, 1.87 wide. Carapace yellow-green; black longitudinal median band present; cervical and radial furrows faint. Ocular area black, with white, orange and black setae. Eyes: AME 0.20, ALE 0.14, PME 0.32, PLE 0.30; AME–AME 0.14, AME–ALE 0.04, PME–PME 0.31, PME–PLE 0.35. Clypeus height 0.25. Chelicerae pale green; promargin with 3 teeth, retromargin with 2 teeth. Maxillae and labium faint yellow. Sternum pale yellow with black markings. Leg measurements: I 14.64 (3.90, 5.02, 3.62, 2.10); II 13.92 (3.73, 4.78, 3.44, 1.97); III 12.23 (3.41, 4.04, 3.07, 1.71); IV 16.85 (4.40, 5.22, 4.79, 2.44). Leg formula: 4-1-2-3. Opisthosoma long-oval; dorsum grayish-black with light brown maculations; cardiac mark elongated, paired lateral stripes distinct; venter center grayish-yellowish brown, with scattered grayish-black patches laterally.

***Palp*** (Figs 1C–F, 2). Terminal apophysis twisted distally, ending in elongated digitiform process curved retrolaterally and ventrally; median apophysis cymbiform, with acuminate apex bent ventrolaterally; embolus filiform, originating from median apophysis base, initially curved dorsally then descending sharply.

**Female** (Fig. 1B): Total length 8.53. Carapace 4.22 long, 3.67 wide; opisthosoma 4.43 long, 2.85 wide. Carapace tawny; black longitudinal median band present; cervical and radial furrows faint. Ocular area as in male. Eyes: AME 0.22, ALE 0.15, PME 0.35, PLE 0.34; AME–AME 0.15, AME–ALE 0.06, PME–PME 0.35, PME–PLE 0.43. Chelicerae brownish-yellow with black stripes; promargin with 3 teeth, retromargin with 2 teeth. Maxillae faint yellow, labium yellow. Sternum yellow with black markings. Leg measurements: I 12.19 (3.43, 4.27, 2.78, 1.71); II 11.52 (3.35, 3.99, 2.55, 1.63); III 10.79 (3.12, 3.61, 2.54, 1.52); IV 15.99 (4.35, 4.99, 4.47, 2.18). Leg formula: 4-1-2-3.

***Epigyne*** (Figs 1G, H, 3). Median septum inverted T-shaped; depression present near insertion pore. Copulatory duct extremely long, spirally coiled; spermatheca rod-shaped; fertilization duct slender, curved.

### ﻿Molecular phylogeny

*Chicosa cinnameopilosa* comb. nov. and all analyzed species of the subfamily Pardosinae cluster into a clade, and it is sister to *Pardosa* + *Wadicosa* (Fig. 4).

## ﻿Discussion

*Chicosa cinnameopilosa* was originally placed in *Tarentula* (Schenkel, 1963), with subsequent revisions by Šternbergs (1981) and Li and Chen (1982), until its monospecific status was confirmed by Marusik et al. (2000). Based on autapomorphies in reproductive structures and phylogenomic evidence, we hereby establish the new genus *Chicosa* gen. nov.

The male palp exhibits a well-developed, hook-shaped terminal apophysis (TA); a slender, sinuous, basally originating embolus (EM); and an undivided, cymbiform median apophysis (MA). The female epigyne possesses an inverted T-shaped median septum (MS) and extremely long, spirally coiled copulatory ducts (CD). These traits differ markedly from all known *Alopecosa* species, particularly the unique configuration of the CD.

Although Yin et al. (1997, 2012) noted convergences in cymbial morphology with *Pardosa*, the new genus is readily distinguished by its basally originating EM (vs. non-basal), hook-shaped TA (vs. dentiform), and spirally coiled CD (vs. short and straight). Furthermore, *Chicosa* differs fundamentally from other available genera: from *Pardosops* Roewer, 1955 (short and robust MA; two spinose TAs); *Acroniops* Simon, 1898 (two-eyed anterior row); *Chorilycosa* Roewer, 1960 (recurved anterior eye row; two spines on TA); and *Leimonia* C. L. Koch, 1847 (strongly recurved and wider anterior eye row).

The unique combination of genitalic and ocular characters precludes its assignment to any known lycosid genus, supporting the establishment of a new genus to reflect its evolutionary distinctness.

## Supplementary Material

XML Treatment for Chicosa

XML Treatment for Chicosa cinnameopilosa

## ﻿Additional information

### Use of AI

No use of AI was reported.

### Author ORCIDs

Ze-Hong Tao https://orcid.org/0009-0006-5364-4127

Dan Fu https://orcid.org/0000-0002-9092-4180

Chang-Jun Wu https://orcid.org/0009-0007-3557-1240

Yang Wang https://orcid.org/0009-0004-6748-5548

Li-Juan Liu https://orcid.org/0009-0009-0410-5858

Yu-Fa Luo https://orcid.org/0000-0002-0645-8712

### Data availability

All of the data that support the findings of this study are available in the main text or Supplementary Information.

## Supplementary materials

Supplementary material 1 Samples used in this study Data type xlsx https://binary.pensoft.net/file/1463995This dataset is made available under the Open Database License (http://opendatacommons.org/licenses/odbl/1.0/). The Open Database License (ODbL) is a license agreement intended to allow users to freely share, modify, and use this Dataset while maintaining this same freedom for others, provided that the original source and author(s) are credited.Ze-Hong Tao, Dan Fu, Chang-Jun Wu, Yang Wang, Li-Juan Liu, Yu-Fa Luo

Supplementary material 2 The concatenated *COI*, 12S and 16S dataset of 329 Lycosidae species Data type fas https://binary.pensoft.net/file/1463996This dataset is made available under the Open Database License (http://opendatacommons.org/licenses/odbl/1.0/). The Open Database License (ODbL) is a license agreement intended to allow users to freely share, modify, and use this Dataset while maintaining this same freedom for others, provided that the original source and author(s) are credited.Ze-Hong Tao, Dan Fu, Chang-Jun Wu, Yang Wang, Li-Juan Liu, Yu-Fa Luo

Supplementary material 3 The concatenated *COI*, 12S and 16S dataset of 85 Lycosinae and Pardosinae species Data type fas https://binary.pensoft.net/file/1463997This dataset is made available under the Open Database License (http://opendatacommons.org/licenses/odbl/1.0/). The Open Database License (ODbL) is a license agreement intended to allow users to freely share, modify, and use this Dataset while maintaining this same freedom for others, provided that the original source and author(s) are credited.Ze-Hong Tao, Dan Fu, Chang-Jun Wu, Yang Wang, Li-Juan Liu, Yu-Fa Luo
